# Humanities for medical students: essential to their cultural competence and patient-engaged practices of care

**DOI:** 10.15694/mep.2018.0000202.1

**Published:** 2018-09-07

**Authors:** Phillipa Malpas, Tanisha Jowsey

**Affiliations:** 1 University of Auckland

**Keywords:** critical reflection, humanities, social, medical students, medicine

## Abstract

This article was migrated. The article was marked as recommended.

Over the past 50 years, advances in medical technology have revolutionised the medical landscape. The ways in which patients, their families, and health professionals connect and interact together in the medical endeavour have also changed. At the same time as advances in medical technology resulted in tremendous gains for society, criticism began emerging that the patient as a whole person had been overshadowed by the disease the person had. The interface of medicine and the humanities concerns itself with our relationships with others, the ways in which we confront challenges to our mortality, how we understand human behaviour, and the meanings we give to situations within the medical context. In this paper we outline the genesis of a medical humanities programme at the University of Auckland, and how the programme has advanced over the past twenty years. The programme promotes important social aspects of medicine such as cultural competence and person-centred engaged care. It also encourages students to improve their academic and reflective writing skills. We discuss how one course within the programme promotes student cultural awareness of - and responsiveness to - Maori people and Deaf people. Specific attention is paid to current leading pedagogy, as well as how the use of space and language contribute to student learning. We conclude that medical humanities remains an essential and valued element in medical curricula.

## Introduction

*‘Doctors need humanistic skills. Perhaps more than at any time in the past, we need doctors who are able to respond sensitively and helpfully to their patients’ emotional needs’* ( Grant, 2002).

Over the past 50 years, advances in medical technology have revolutionised the medical landscape. The advent of new fields such as genomics, epigenetics, and bioengineering emerged from the pioneer work taken in understanding the human genetic code ( Cavalli-Sforza and Bodmer, 1999). The use of robotics in surgery, simulation training, and mobile wireless implantable devices have enabled better patient care by saving lives, increasing access to care and improving quality of life ( Bonato, 2010; McGaghie *et al.*, 2010; Russell, 2010). Health professionals can now connect with their patients via digital technologies such as tele-health ( Brodie *et al.*, 2000; Darkins *et al.*, 2008).

The ways in which patients, their families, and health professionals connect and interact together in the medical endeavour have also changed. Patients are encouraged to ask questions, share in decision-making ( Prey *et al.*, 2013), and think about the kinds of medical treatment and care they prefer at the end of life when their capacity to make decisions may have diminished ( Sudore and Fried, 2010). Health professionals are expected to respect the values of their patients and take seriously their preferences in medical decision-making ( Barry and Edgman-Levitan, 2012). How doctors meet *patients as persons* is as important as the biomedical approach, perhaps even more so.

At the same time as advances in medical technology resulted in tremendous gains for society, criticism began emerging that the patient as a whole person had been overshadowed by the disease the person had. William Osler had been perceptive to the whole person, noting more than one hundred years ago that, “it is much more important to know what sort of a patient has a disease than what sort of a disease a patient has”( John, 2013).To put this another way, at the heart of medicine lies human activity and humanity ( Evans and Greaves, 1999; Chiapperino and Boniolo, 2014). The interface of both medicine and the humanities is concerned with our relationships with others, the ways in which we confront challenges to our mortality, how we understand human behaviour, and the meanings we give to situations within the medical context. Harnessing the insights and perspectives from a number of humanities disciplines illuminates our understanding of medicine’s nature and goals, and it is this focus that offers medical students a profoundly valuable perspective on what it is to be human - both from the patient’s viewpoint and their own - in the search for competent and appropriate responses in professional health care. The wider perspectives and meanings around health, illness, disease, normality and abnormality, compassion, anxiety, grief, human needs and interests, healing, suffering, birth, living and dying, and death, are also central in many of the humanities disciplines. Many medical schools incorporate the humanities into their curricula recognising the contribution, perspectives and insights that the humanities brings to the practice of medicine ( Bourgois, 2003; Hackler, 2003; Shafer, 2003).

In this paper we outline the genesis of the medical humanities programme at the University of Auckland, and how the programme has advanced over the past twenty years. We discuss how one course - Medical Anthropology - promotes student cultural awareness of, and responsiveness to, people of diverse cultures such as Maori people and Deaf people. Specific attention is paid to current leading pedagogy, as well as how the use of space and language contribute to student learning. We include some examples of student’s work to illustrate their learning about cultural competence and patient-engaged practices of care.

## Establishment of the Medical Humanities programme at Auckland University

In the early 1990’s after a series of interfaculty committee discussions involving representatives from a number of arts disciplines as well as medical specialities, two fundamental principles emerged on which all decision making would lie in the introduction of the humanities into the medical curriculum. First, “all courses would be taught only by people accredited and approved by the discipline”; and second that the courses “should be relevant to medicine, resulting in improved patient care”( Grant, 2002).

The requirement that teachers would be accredited and approved by the discipline underpinned the importance of ensuring academic rigour and the critical analysis and discourse of ideas; a central tenet of humanities disciplines. Ensuring that courses were relevant to medicine and therefore to medical students, reflected the importance of student engagement as well as how the humanities can contribute to understanding the human condition, and thus to improving how doctors respond to their patients. A fundamental goal of the medical humanities was, and continues to be, cultivating and personalising the relationship between the patient and the health professional.

Goals of our Medical Humanities programme:

1. To foster reflection and deliberation among medical students on the human condition (loss, love, dying, grief, anxiety, life and death)
2. To gain insights into the experiences of illness, disease and injury
3. To encourage effective communication within science and medicine
4. To develop critical thinking to deepen and enhance the work of doctors, and to stimulate creativity and reflection
5. To encourage the doctor to incorporate his or her work into a wider relationship with other areas of knowledge
6. To promote and encourage scholarly work at the interface of medicine (science) and the humanities (arts).

In 1996 year 2 medical students could study humanities courses in elective papers. Over five years these were further developed and refined as a result of student and peer evaluations and assessments. In 2000, the medical humanities programme became compulsory for year 3 students who were required to select one course from eight on offer. They spanned the disciplines of law, philosophy, sociology, political studies, classics, English literature, history and art. Classes were small (around 14-18 students) to facilitate discussion and debate, something more challenging to achieve in larger classes of 150 students. Courses span a 12 week semester with classes running for 3 hours per week.

Almost twenty years later the medical humanities programme continues to thrive with 14 courses offered in 2018 to year 3 medical students across a wide range of humanities disciplines, including; gender studies, research ethics, comparative literature, theology, music, philosophy, law, history, classics, art history, education, anthropology, heredity, development and evolution, and English, writing and drama studies ( table 1). Students are asked for their four preferences, with almost all students studying one of their course selections for a semester (12 weeks).

**Table 1.** Medical humanities programme at the University of Auckland

Humanities discipline	Brief summary
Gender studies	This course considers the medical needs, health and wellbeing of people who do not fit dominant definitions of ‘normal’ bodies, identities or desires.
Research	This course provides selected students with the opportunity to explore the value of research in an area of interest to them. Students will come to understand the role that research activities (fundamental or applied) play in medical practice and health policy.
Comparative literature	Through the study of literary texts about medicine and a review of the many kinds of medical metaphors and narratives, we will attempt to articulate some of the connections between medicine and literature.
Theology	In this course, we will examine some of the beliefs and practices of spirituality and religious traditions, thinking about the different ways that can be associated with health and healing in medical and mental health contexts.
Music	This course examines some of the critical physical and psychological health issues musicians encounter when preparing for performances and when performing.
Philosophy (Ethics)	This course will focus our philosophical lens on a number of contemporary topics within medicine with compelling ethical dimensions.
Law	The aim of this course is to introduce students to the important role the law plays in the practice of medicine and the delivery of health care.
History	This course will explore aspects of the history of western bio-medicine since the 19 ^th^ century using examples and the historical literature from Britain, the USA and New Zealand.
Classics	The role of medicine within contemporary religious, philosophical, ethical and scientific perspectives will allow students to understand the wider context of medicine in the ancient world.
Art History (In 2018 two different courses were run within the discipline of Art History)	This course aims to examine in depth artworks that raise ethical and philosophical issues pertaining to the body. This course examines issues around documentation and various ways artists communicate to viewers their preoccupations with information about human life, with particular attention drawn to discrepancies of power
Education	We will review key aspects of teaching theory and practice that will inform and enable you to teach more effectively
Anthropology	Students will learn **how to identify**and **critically appraise** messages in literature, images and films anthropologically.
English, writing and drama studies	The course is structured with weekly units on plays, films, television, and applied theatre. There is a strong focus on text analysis, looking at the narrative, thematic and ethical questions about medicine that they raise.
Heredity, development and evolution	In this course we will explore how, in the nineteenth century, heredity came to be understood as a fixed quality and explained in genetic terms. We will discuss what it meant for the understanding of health, disease, human diversity and evolution.

Although the courses are designed and developed for medical students from the specific humanities disciplines, the assessment criteria is reasonably consistent across all courses to ensure fairness and equity. Requirements include: an oral seminar given by each student, a research essay or other work requiring a strong research focus (for instance, a critical review of an academic paper), and a small mark given for engagement and contribution in class. A medical humanities’ prize is awarded each year for the best overall piece of work submitted by a student. Many of these have been published ( Bollen, 2011; Holford, 2012; Jiang, 2012; Mohd Slim, 2013).

Courses within our programme are regularly appraised by students which includes an evaluation of the lecturer(s) leading the course, and of the course content. Overall, course and lecturer evaluations have consistently demonstrated high student satisfaction. These provide valuable and necessary feedback which assist in directing and promoting change, and in improving how we teach and instruct within the classroom.

## Humanities encourage student critical reflection, written skills development and publication

The written assessments for the humanities courses offer students an opportunity - and usually the first one they have had since leaving high school - to practice and hone their formal and creative writing skills. This opportunity supports them to be successful in their future academic publishing endeavours (see for example, Bollen, 2011; Holford, 2012; Jiang, 2012; Mohd Slim, 2013). Teachers of the Comparative Literature course - Unexamined Metaphors, Uncharted Stories - create an annual anthology of their students’ poetry (written during the course) and distribute it amongst their students upon the course conclusion.

As part of their contribution to the Medical Anthropology class, students work to create evidence-based summaries of key terms that are then moderated by the course co-ordinator before being submitted to biomedical Wikipedia pages. An example of this is the Wikipedia page concerning biomedicine, whereby students have now anonymously contributed a paragraph concerning how biomedicine is viewed in social sciences ( Wikipedia, 2018).

The humanities courses, which are offered to year 3 students, inform student ideas and writings in their following years. Each year from year 3 onwards students are required to write and submit a Personal and Professional Skills Portfolio for assessment. It is through the Portfolio that students are to demonstrate critical reflection on their experiences and on the kind of doctor they want to be. Students often bring their experiences and lessons from the year 3 humanities courses to bear on their Portfolios. In 2017 a collection of 102 poem and art images that students submitted to their Portfolios were published, showcasing their insights concerning multiple humanities domains such as professionalism, gender, ethics, history, doctor-patient communication, and cultural competence ( Jowsey, 2017). A rich exemplar of how these all come together in the Portfolio comes from Monica Pritchard’s 2016 poem *Do not resuscitate*( Jowsey, 2017).

### DO NOT RESUSCITATE

*(Doctor says)*

Sir, you are dying

prayers cannot not save you now

miracles are merely fairytales told in church

giving false hope

to the weak.

So when your heart decides to fail

let us not jump up and down on your chest

for you will never be the same again

do not confuse resuscitation with resurrection.

*(Patient replies)*

Doctor, I am dying

Pills and machines cannot save me now

Trials are merely hypothesis drafted in textbooks

giving false hope

to the ill.

So when God decides to call my name

do not bother jumping up and down on my chest

for I will be long gone by then

do not confuse body with soul.

Monica Pritchard, Year 4, as Cultural Competence

Jowsey, T. (ed.) 2017. *Medicine Reflections.* Auckland: Compassion Publishers: 66

## Medical Anthropology course development

New Zealand provides a wonderful melting pot of intersecting cultures and ethnicities; making it a multicultural cosmopolitan hub in the South Pacific region. This brings several challenges to health care practitioners in terms of culturally-specific issues concerning practices of patient health-care seeking and clinical practices of care ( Jowsey, 2018).

Anthropology is the study of human culture ( Rapport, 2014). In 2015, TW began designing a course of human health cultures to sit within the humanities programme. She teamed with educationalist and cultural historian, Pauline Cooper-Ioelu, to support the course design with strong constructive alignment and innovative student-engaged learning methods. The course has been offered for three consecutive years, each time being refined in response to student feedback and evaluations.

We begin with an exploration of culture and how we might come to think about people’s ideas and practices anthropologically. Students are introduced to the concept of an ‘anthropological lens’, which they are encouraged to use in exploring how cultural boundaries are broached and bridged between health care practitioners and patients/consumers/health service users. This begins their journey towards increasing cultural competence as future medical professionals.

**Image 1.**
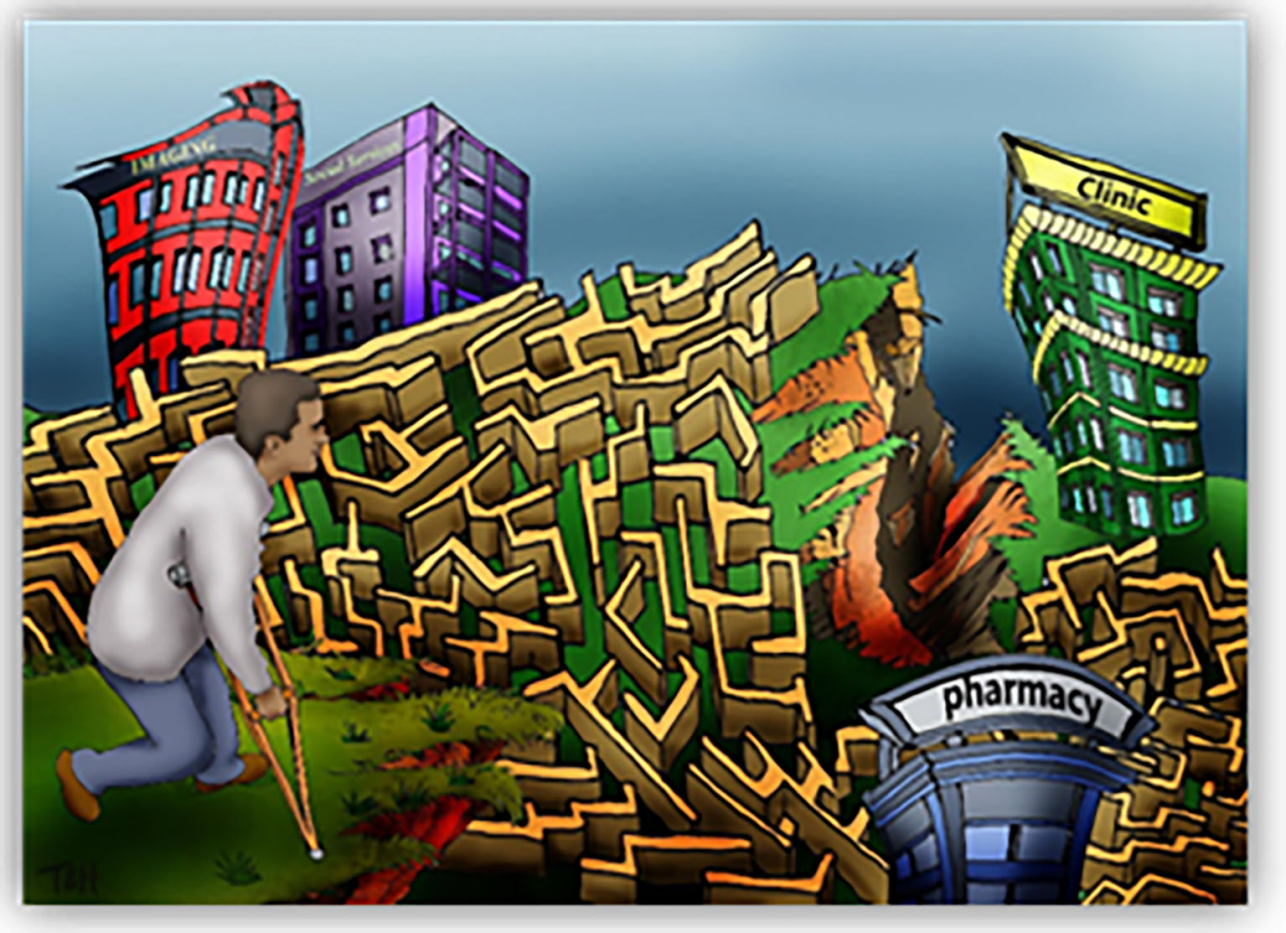
*Cultural Maze* by T Jowsey, 2016

We unpack terms such as culture, ethnicity, cultural safety, cultural boundaries, agency, structure, power, discourse and taboos. What cultural boundaries do medical students need to know about to provide culturally-safe and effective care? Is there such a thing as hospital culture? How could we recognise it? Are power hierarchies in medicine measurable phenomena that can inform medical practice? And if so, what bearing does this have on patient safety? These questions and others are addressed through 12 student-engaged teaching sessions. We use basic components of a flipped classroom format and cooperative learning theory to promote engagement( Johnson, Johnson and Smith, 1998; Kenwright *et al.*, 2016). The teaching sessions are all designed to optimise student engaged learning through in-class activities such as debates, poster design, small-group work and whole-class discussions. The learning is supported with original visual resources that we have developed for the course (see for example, image 1).

Since its inception in 2015, various spaces have featured as part of the teaching design. Some iterations of the course have involved teaching on a marae (traditional Maori meeting ground) in the Whare Nui (Main Meeting house where cultural knowledge and wisdom is traditionally passed on) where students have the rare privilege of hearing from invited Maori elders about their beliefs and practices concerning health care. The course has included walking guided art tours of our local hospital and guided poetry writing on a grassy hill outside the medical school. Changing the learning spaces, and the ways in which students experience medical spaces is an intentional anthropological and teaching technique we use to promote cultural competence and engaged learning.

Language is also a strong cultural aspect of our attention in the course. We look at the three official languages of New Zealand: English, te reo Maori, and New Zealand Sign Language (NZSL). In week one of the course students are asked to compile a list of words (in English) that they would like to know in Maori and NZSL, which they anticipate will be of value to them as future doctors. Some words that students have previously chosen include: hello, doctor, nurse, pain, where, sick, hospital, water, nil by mouth, and medicine/pills. Each week thereafter the class begins with students learning 10 words in Maori and in NZSL, and reviewing words from previous sessions. We also cover additional words that we suggest may be of benefit to students, such as: you’re welcome, permission, and family. The course concludes with a Deaf guest teacher, with an NZSL interpreter running a session about Deaf culture and health. By the end of the course students have a strong understanding of the intersections between culture and medicine, they have specific applicable knowledge about Maori and Deaf cultures, and they demonstrate increasingly broad cultural competence. In course evaluations and in the year 4 & 5 portfolios students have identified these kinds of learning opportunities as invaluable to shaping their medical ideas and practices.

## Conclusions

“The arts are the vehicles through which human beings articulate the meanings of their lives” ( Calman and Downie, 1996).

Over the past twenty years the medical humanities programme at the University of Auckland has developed from students electing to study in a course alongside humanities students, to a comprehensive programme that offers bespoke courses to medical students. Courses have been created to satisfy and complement the growing demand for expertise knowledge in emerging areas such as gender studies, and in medical education where the doctor is both a leader and teacher (see Table 1), reflecting the diverse nature of the health professional’s role in contemporary society.

At the heart of our medical humanities programme is recognition that the medical arena is constantly changing. The ways in which we now communicate, engage, and interact with each other have undergone fundamental changes that early medical thinkers and pioneers like William Osler and others could have scarcely imagined. But central to their vison was recognition of the importance and significance of the whole person not just their illness, injury, or disability. That vision is central to our medical humanities programme and is poignantly illuminated in the following poem by one of our medical students.

### I MET A MAN TODAY

I met a man today

He is old and I am young

He is dying, I am well.

He is lost

So am I.

I met a man today.

I held his hand, I felt his pain.

Tears roll down his writhing, broken body. Screams of anguish, confusion, desperation.

His body fighting, his eyes empty. Part of him already gone.

His body missed the memo.

He gasps for breath, body shaking. His arm snaps outwards, a pale blur.

My arm exposed, fingers squeezing, nails sinking deep,

His strength takes my breath.

One last try. I can see it in his face. Why will she not help me?

The sharpness of the pain feels odd, far away, detached.

The heavy aching in my chest does not.

My words of comfort comfort no one.

I met a man today.

It was not peaceful, it was not quick.

His family gone. His nurse too busy.

Just sit with him until he passes. Then come and join us on the ward round.

Jowsey, T. (ed.) 2017. *Medicine* *Reflections.* Auckland: Compassion Publishers: 37

### Take Home Messages

• The University of Auckland has been running a successful medical humanities programme for more than twenty years.
• Humanities education for medical students is critical to shaping their cultural competence and patient-engaged care.
• Humanities education scaffolds students towards successful academic writing and publication.

## Notes On Contributors

PJM is coordinator of the medical humanities programme, and Associate Professor of Clinical Medical Ethics at the University of Auckland.

TJ is a medical and visual anthropologist, and lecturer in clinical education at the University of Auckland.
