# Supplementary material for: Humanities for medical students: essential to their cultural competence and patient-engaged practices of care
Source: MedEdPublish (2016). 2018 Sep 7;7:202. [Version 1] doi: 10.15694/mep.2018.0000202.1 (PMC10701830; doi:10.15694/mep.2018.0000202.1)
Supplement: Peer review history

# Reviewer report: Reviewer response for version 1

**DOI:** 10.21956/mep.19655.r29319

**Published:** 2018-09-19

**Authors:** Jonathan McFarland

**Affiliations:** 1 Sechenov University, Moscow

This review has been migrated. The reviewer awarded 5 stars out of 5 This is a fascinating paper, in which the authors draw on their more than 20 years of experience of teaching medical humanities at the University of Auckland through their establishment of a Student Selected Component. They highlight how the vision central to pioneers of medical education such as William Osler, who recognised the importance and significance of the whole person and not just their illness, injury or disability, is now more critical than ever. It describes brilliantly how the teaching of humanities has progressed through the years, transforming from an elective to compulsory. Whenever we discuss the importance of how to implement the humanities into the curriculum then I believe that we could look to this paper for guidance. For me, just the extensive range of topics is worthy of mention. I would also like to mention the strong cultural work done with the three official languages in New Zealand: English, te reo Maori and New Zealand Sign Language. Laudable indeed, and for me one of the highlights of this Theme issued can clearly be seen in this paper; the importance of the “local” context can be a guide for the “global”, that is to say, it is so important to see what is happening locally around the globe, learn from it and then use it in your particular setting. However, I would not be true to myself if I did not mention that the poignant use of poetry, written by the medical students was special, and I would be extremely interested to see the 2017 collection. It is a rich and powerful paper that I strongly recommend to be read by all with an interest in this topic.

Reviewer Expertise:

NA

No decision status is available

# Reviewer report: Reviewer response for version 1

**DOI:** 10.21956/mep.19655.r29321

**Published:** 2018-09-15

**Authors:** BALAJI ARUMUGAM

**Affiliations:** 1 TAGORE MEDICAL COLLEGE AND HOSPITAL

This review has been migrated. The reviewer awarded 5 stars out of 5 It’s awesome to know that the humanities programme was there since 20 years in Auckland. The article expresses how humanities encourage student critical reflection, writing skills and publication. The goals of the medical humanities porgramme narrated were evidently achieved. Congrats to the establishment and successful implementation of such a great programme. Curriculum developers should look into such examples. I have thoroughly enjoyed reading this paper. Thanks to the author.

Reviewer Expertise:

NA

No decision status is available

# Reviewer report: Reviewer response for version 1

**DOI:** 10.21956/mep.19655.r29320

**Published:** 2018-09-07

**Authors:** P Ravi Shankar

**Affiliations:** 1 American International Medical University

This review has been migrated. The reviewer awarded 5 stars out of 5 I enjoyed reading this well-written paper about the medical humanities (MH) program at the University of Auckland. I am happy to know that the program is over 20 years old. The authors emphasize the important of looking at a person with an illness and not at an illness within a person. I agree that in today’s modern medical practice with its emphasis on efficiency and specialization the patient perspective may often not be considered. The authors provide a clear description of the various choices students have with regard to MH. The examples of works by students add to the strength of the article. I enjoyed the two poems by the students and Table 1 which provides a brief description of the various courses/subjects offered during the MH program. The description of the medical anthropology course was especially interesting. All educators with an interest in MH will find this article to be of interest.

Reviewer Expertise:

NA

No decision status is available

# Reviewer report: Reviewer response for version 1

**DOI:** 10.21956/mep.19655.r29318

**Published:** 2018-09-07

**Authors:** Trevor Gibbs

**Affiliations:** 1 AMEE

This review has been migrated. The reviewer awarded 5 stars out of 5 This was a very good paper to read, describing one school's approach to the teaching of the Humanities. I thought that it was very well written , with a very powerful message that relates to the ability of the humanities to address very important qualities within our developing students, as well as addressing the issue of cultural diversity and patient connectivity.I loved reading the poetry provided by the students and if this is a typical example of their work then more power to them. I have to say that reading their examples brought a lump to my throat- they were certainly very touching and were a great way of showing their real feelings and thoughts about patient care.Given the longevity of this course then I feel that the school and these authors are in an ideal position to look at the longer term effects of this course - I feel that this what is needed to put the icing on the humanities cake.All those involved in curriculum planning should read this paper.

Reviewer Expertise:

NA

No decision status is available
